# Asthma control and care among six public health clinic attenders in Malaysia: A cross‐sectional study

**DOI:** 10.1002/hsr2.1021

**Published:** 2023-05-02

**Authors:** Norita Hussein, Su May Liew, Nik Sherina Hanafi, Ping Yein Lee, Ai Theng Cheong, Sazlina Shariff Ghazali, Karuthan Chinna, Yong Kek Pang, Asiah Kassim, Richard A. Parker, Jürgen Schwarze, Aziz Sheikh, Hilary Pinnock, Ee Ming Khoo, the RESPIRE Collaborators

**Affiliations:** ^1^ Department of Primary Care Medicine, Faculty of Medicine Universiti Malaya Kuala Lumpur Malaysia; ^2^ UM eHealth Unit, Faculty of Medicine Universiti Malaya Kuala Lumpur Malaysia; ^3^ Department of Family Medicine, Faculty of Medicine and Health Sciences Universiti Putra Malaysia Seri Kembangan Malaysia; ^4^ Faculty of Business and Management UCSI University Kuala Lumpur Malaysia; ^5^ Department of Medicine, Faculty of Medicine Universiti Malaya Kuala Lumpur Malaysia; ^6^ Kuala Lumpur Women and Children Hospital, Ministry of Health Kuala Lumpur Malaysia; ^7^ Edinburgh Clinical Trials Unit, Usher Institute The University of Edinburgh Edinburgh UK; ^8^ NIHR Global Health Research Unit on Respiratory Health (RESPIRE), Usher Institute The University of Edinburgh Edinburgh UK; ^9^ Child Life and Health, Centre for Inflammation Research The University of Edinburgh Edinburgh UK

**Keywords:** asthma, asthma control, primary care, risk factors

## Abstract

**Background and Aims:**

Asthma is common in Malaysia but neglected. Achieving optimal asthma control and care is a challenge in the primary care setting. In this study, we aimed to identify the risk factors for poor asthma control and pattern of care among adults and children (5–17 years old) with asthma attending six public health clinics in Klang District, Malaysia.

**Methods:**

We conducted a cross‐sectional study collecting patients’ sociodemographic characteristics, asthma control, trigger factors, healthcare use, asthma treatment, and monitoring and use of asthma action plan. Descriptive statistics and stepwise logistic regression were used in data analysis.

**Results:**

A total of 1280 patients were recruited; 85.3% adults and 14.7% children aged 5–17 years old. Only 34.1% of adults had well‐controlled asthma, 36.5% had partly controlled asthma, and 29.4% had uncontrolled asthma. In children, 54.3% had well‐controlled asthma, 31.9% had partly controlled, and 13.8% had uncontrolled asthma. More than half had experienced one or more exacerbations in the last 1 year, with a mean of six exacerbations in adults and three in children. Main triggers for poor control in adults were haze (odds ratio [OR] 1.51; 95% confidence interval [CI] 1.13–2.01); cold food (OR 1.54; 95% CI 1.15–2.07), extreme emotion (OR 1.90; 95% CI 1.26–2.89); air‐conditioning (OR 1.63; 95% CI 1.20–2.22); and physical activity (OR 2.85; 95% CI 2.13–3.82). In children, hot weather (OR 3.14; 95% CI 1.22–8.11), and allergic rhinitis (OR 2.57; 95% CI 1.13–5.82) contributed to poor control. The majority (81.7% of adults and 64.4% of children) were prescribed controller medications, but only 42.4% and 29.8% of the respective groups were compliant with the treatment. The importance of an asthma action plan was reported less emphasized in asthma education.

**Conclusion:**

Asthma control remains suboptimal. Several triggers, compliance to controller medications, and asthma action plan use require attention during asthma reviews for better asthma outcomes.

## 1 INTRODUCTION

Asthma is a major and increasing health concern in Asia.^1^ In Malaysia, the prevalence is estimated at 8.9%–13.0% in children^2^, ^3^ and 6.3% in adults.^4^ Although asthma is a preventable condition, the prevalence of poorly controlled asthma is still high and has a substantial impact on the use of public health care resources and health expenditure.^5^ The Malaysian National Health and Morbidity Survey reported that every year, 20% of adults with asthma visited the emergency department for acute exacerbations, and 10% of these were admitted.^6^ Another local study on 311 children with asthma reported about half of them had poor control and that a third had emergency visits for asthma in the previous 1 year.^2^ The high cost of asthma management was due mainly to hospitalization and medication.^7^ Mortality for asthma in Malaysia has also increased since 1990 by an average of 0.6% a year, which is preventable.^8^ The main aim of asthma management is to achieve and maintain good asthma control. Primary care plays a pivotal role in providing optimal asthma care, but currently several limitations exist. Most patients’ knowledge about asthma is poor, with reports stating limited provision of education on the disease and its treatment.^2^, ^9^ Effective medications are available but local qualitative study has reported suboptimal use of inhaled controller medications amongst children with asthma and most used complementary and alternative medication (CAM) for self‐treatment instead.^10^ Despite overwhelming evidence for supported self‐management,^11^ the uptake of education on asthma and self‐management skills in Malaysia is low.^9^

Determining risk factors for poor asthma outcomes can potentially help target the provision of asthma management in the primary care settings. Our overarching aim was to define a population at greater risk of poor control; to describe the monitoring and treatment, and to report on the provision of asthma care. In this study, we intend to establish a cohort of patients to improve our understanding of the complex factors related to disease burden and to inform the development of quality improvement initiatives in our primary care settings.

## 2 METHODS

### 2.1 Setting

This cross‐sectional study was carried out between July 2019 and January 2020 in six public health clinics in the Klang District, Malaysia. The Klang District was chosen because it is a densely populated district with a mix of rural, suburban and urban settings.^12^, ^13^, ^14^, ^15^ The six chosen health clinics cater for a low‐ and middle‐income population. They are government‐funded clinics in which patients pay a nominal fee of MYR 1 (USD 0.24) per visit for consultation, investigation, and treatment.^15^ Senior citizens, children, and government employees receive free treatment. The prevalence of asthma in Selangor was reported to be 5.9% in 2011.^16^

### 2.2 Participants and sampling

Sample size was calculated to enable assessment of a proposed improvement initiative using the G*Power 3.1 software using estimates from a meta‐review of supported self‐management of asthma.^17^ A total of 766 patients were required to have an 80% chance of detecting significance at the 5% level a decrease in the primary outcome measure (proportion with an unscheduled consultation) from 22% in the control group to 14% in the experimental group. To allow for an estimated dropout rate of 40%,^18^ we recruited 1280 patients aged 5 years and above with physician‐diagnosed asthma and/or receiving asthma treatment in the previous year. Patients with respiratory symptoms (e.g. cough, breathlessness, wheezing) due to respiratory infections without underlying asthma, chronic obstructive pulmonary disease (COPD), congenital heart disease, gastroesophageal reflux, heart, liver or renal failure, active life‐threatening malignancy and those receiving palliative care were excluded.

### 2.3 Recruitment

These health clinics did not have registers of people with asthma; hence patients were recruited during their routine visits to the clinic. We obtained written informed consent from patients upon recruitment. For children aged 7–17, age‐appropriate assent and parental consent were sought. For children aged below 7, only parental consent was sought.^19^ All patients who fulfilled the inclusion criteria and consented, were screened with handheld spirometry (if not contraindicated) using the Vitalograph® micro spirometer.^20^ Those with an FEV_1_/FVC ratio below 0.7, were asked to return within 2 weeks to perform formal bronchodilator reversibility testing with the ndd EasyOne Air spirometer.^21^ We included patients who demonstrated reversibility (defined as having an FEV_1_ value increase by 200 ml or more or an increase of 12% and above from baseline).^22^ If the patient did not demonstrate reversibility they were classified as having COPD and were excluded. Patients who had achieved an FEV1/FVC ratio of 0.7 and above (i.e., normal spirometry) were included in the study based on clinical diagnosis. Patients who were unable to perform the handheld spirometry appropriately or whose spirometry results were inconclusive were invited to a repeat assessment within 2 weeks to clarify the diagnosis.

### 2.4 Data collection

There were two types of data collected: data from face‐to‐face questionnaires and clinical assessments. The questionnaires included sociodemographic characteristics, healthcare use, and related payment, current asthma status, comorbidities, asthma treatment, use of asthma action plan (AAP), asthma education, and EuroQol‐Visual Analogue Scale (EQ‐VAS). EQ‐VAS is a tool to self‐rate overall measure of health status on a vertical scale of 0–100 (worst to best)^23^ at the time of recruitment. For children, the questionnaires were usually answered by their parents or carers, although a few older children completed the questionnaire themselves with the help of their parents. Clinical assessments included blood pressure and peak flow measurements. Asthma control was evaluated using GINA assessment of asthma control (GINA 2017) and was categorized into well‐controlled, partly controlled, and uncontrolled.^22^ Data were also retrieved from patients’ medical records to ensure clarity on the reason of visit at the point of recruitment; these include asthma diagnosis with spirometry measurement (if available), degree of asthma control, previous peak flow measurements, education on inhaler technique, and prescribed treatments.

### 2.5 Ethics approval and informed consent

Ethical approval was obtained from the National Medical Research Ethics Committee, Ministry of Health, Malaysia (NMRR‐18‐2707‐42719) and the sponsor, the Academic and Clinical Central Office for Research and Development, University of Edinburgh, United Kingdom (AC19040). A written consent indicating the purpose of the study and the participant's right to withdraw at any time without any obligation towards the study team was obtained from each participant.

### 2.6 Patient and public involvement (PPI)

Five representatives were involved in the development of the study protocol. They reviewed the protocol and all patient‐related documents which included the questionnaires, participant information sheets, and consent forms.

### 2.7 Statistical analysis

Statistical analysis was done using SPSS version 25.0. Data were summarized using frequencies and percentages for categorical variables or means and standard deviation (SD) for continuous variables. The variables were included as individual explanatory variables in logistic regression models fitted to the GINA outcome variable, which was dichotomized based on the adequacy of asthma control into well‐controlled asthma and poorly controlled asthma (partly controlled and uncontrolled). Variables with a *p*‐value of < 0.25 in this analysis were taken as independent variables for multivariable logistic regression using forward stepwise model selection to ensure no important variables are left out from the final model.^24^, ^25^ This model was used to determine possible factors associated with poor asthma control and variables with a *p*‐value < 0.05 were taken as statistically significant associations with poor asthma control. Odds ratios (OR) with 95% confidence intervals (CI) were reported.

## 3 RESULTS

### 3.1 Sociodemographic profile

A total of 1280 patients were recruited. There were 1092 (85.3%) adults aged ≥ 18 years and 188 (14.7%) children aged 5–17 years. Table 1 shows patients’ sociodemographic characteristics. The majority of adult patients (75.5%) and children's carers (78.7%) were from low‐income households (B40 < USD1196).^25^ Personal savings was the main source of health payment for most patients. Overall, 78 (6.1%) had missing financial data about household income and source of health payment because patients or carers (for children) chose not to complete these questions.

**Table 1:** Sociodemographic profile

	Adults (*N* = 1092)	Children (*N* = 188)
	*n* (%)	*n* (%)
Age		
Mean (SD) years	48.9 ± 15.5	10.7 ± 3.37
Gender		
Male	376 (34.4)	104 (55.3)
Female	716 (65.6)	84 (44.7)
Ethnicity		
Malay	590 (54.0)	147 (78.2)
Chinese	111 (10.2)	10 (5.3)
Indian	374 (34.2)	30 (16.0)
Others	17 (1.6)	1 (0.5)
Level of education		
No formal education	49 (4.5)	11 (5.9)
Kindergarten	0	7 (3.7)
Primary	292 (26.7)	120 (63.8)
Secondary	506 (46.3)	50 (26.6)
Tertiary	245 (22.4)	NA
Born term		
Full term	NA	176 (93.6)
Premature	NA	12 (6.4)
Completed vaccination up to date	NA	187 (99.5)
Occupation		
Not working	419 (38.4)	NA
Retired	132 (12.1)	NA
Working	541 (49.5)	NA
Public sector	63 (12.0)	NA
Private sector	397 (73.4)	NA
Self‐employed	79 (14.6)	NA
Household income^a^		
B40 (low)	824 (75.5)	148 (78.7)
M40 (middle)	178 (16.3)	32 (17.0)
T20 (upper)	18 (1.6)	2 (1.1)
Missing	72 (6.6)	6 (3.2)
Main source of health payment^b^		
Personal savings	667 (61.1)	162 (86.2)
Employment benefits	165 (15.1)	20 (10.6)
Private insurance	56 (5.1)	5 (2.7)
Others	132 (12.1)	0
Missing	72 (6.6)	1 (0.5)
Smoking status		
Current smoker	104 (9.5)	3 (1.6)
Ex‐smoker	158 (14.5)	2 (1.1)
Passive smoker	441 (40.4)	77 (41.0)
		Father‐69 (89.6)
		Mother‐0 (0)
		Siblings‐9 (11.7)
		Others‐8 (10.4)
Obesity category^c^	Body mass index (BMI)	Centile
Underweight	<18.4: 41 (3.8)	<3rd: 23 (12.2)
Normal	18.5‐22.9: 165 (15.1)	3rd‐ 85th: 99 (52.7)
Overweight and obese	≥23.0: 886 (81.1)	>85th: 66 (35.1)
Family history of asthma	683 (62.5)	141 (75.0)
Grandparents	174 (15.9)	59 (31.4)
Parents	388 (33.5)	86 (45.7)
Siblings	334 (30.6)	64 (34.0)
Children	239 (21.9)	NA
Comorbidities		
Hypertension	459 (42.0)	0
Dysplidemia	426 (39.0)	0
Diabetes mellitus	232 (21.2)	0
Gastroesophageal reflux	214 (19.6)	8 (4.3)
Allergic rhinitis	187 (17.1)	36 (19.1)
Obstructive sleep apnea	62 (5.7)	0
Cardiac disorder	76 (7.0)	1 (0.5)
Distance from home to nearest health facility		
Mean (SD) kilometer	3.6 (3.6)	3.4 (3.4)

Abbreviations: MYR, Malaysia Ringgit; *N*, denominator; *n*, numerator; NA, not applicable; SD, standard deviation.

^a^ B40, M40, and T20 refer to the household income classification in Malaysia, categorized as Bottom (low) 40%, Middle 40%, and Top (upper) 20%. B40, M40, and T20 refers to a household income of <MYR4850 (<USD1174.33), MYR4850–10,959 (USD1174.33–2,653.51) and >MYR10,959(>USD2653.51), respectively.^26^

^b^ Personal savings refer to savings from personal and households; employment benefits refer to government, private and social security organization (SOCSO); others include free treatment, charity, nongovernment organization.

^c^ Obesity category based on Malaysian Clinical Practice Guidelines: Management of Obesity. Kuala Lumpur: Ministry of Health, Malaysia, 2004.

### 3.2 Asthma control and related risks

Table 2 summarizes the level of asthma control and related risk profiles. Among 1092 adults, only 34.1% had well‐controlled asthma. Of 188 children, 54.3% were well‐controlled. In the past 1 year, about two‐thirds of patients experienced one or more asthma exacerbations, with a mean of six exacerbations per year in adults and three in children; about half of the adults and children had visits to emergency department; 1 in 10 adults and 1 in 6 children had hospital admissions. Reported main triggers for acute exacerbations were upper respiratory tract infection, indoor dust, haze pollution, cold weather, and food (mainly cold beverages, ice‐cream). About a fifth of both groups reported using complementary alternative medicine (CAM) to treat asthma.

**Table 2:** Asthma control and clinical characteristics

	Adults (*N* = 1092)	Children (*N* = 188)
	*n* (%)	*n* (%)
Asthma control (GINA 2017)^a^		
Well Controlled	372 (34.1)	102 (54.3)
Partly Controlled	399 (36.5)	60 (31.9)
Uncontrolled	321 (29.4)	26 (13.8)
Had asthma exacerbation in the past 12 months	644 (59.0)	121 (64.4)
Frequency, mean (SD)	6.51 (21.0)	3.13 (3.7)
Had visits to emergency department in the past 12 months	548 (50.5)	105 (55.9)
Frequency, mean (SD)	1.7 (3.4)	1.65 (3.1)
Had hospital admission in the past 12 months	117 (10.7)	30 (16.0)
Had used SABA > 2 times/week in the past 1 month	509 (46.6)	46 (24.5)
Reported trigger factors		
URTI	686 (62.8)	122 (64.9)
Indoor dust	773 (70.8)	106 (56.4)
Cold weather	673 (61.6)	107 (56.9)
Air conditioning	375 (34.3)	48 (25.5)
Haze	555 (50.8)	63 (33.5)
Food (cold drinks, ice‐cream)	665 (60.9)	116 (61.7)
Sports/Physical activity	471 (43.1)	61 (32.4)
Extreme emotion	186 (17.0)	13 (6.9)
Animals	183 (16.8)	30 (16.0)
Fumes	199 (18.2)	7 (3.7)
Had used CAM for asthma	201 (18.4)	42 (22.3)
EQ‐VAS mean score	68.4	76.5

Abbreviations: CAM, complementary alternative medicine; EQ‐VAS, EuroQol‐Visual Analogue Scale; GINA, Global Initiative for Asthma; *N*, denominator; *n*, numerator; SABA, short‐acting beta agonist; SD, standard deviation; URTI, upper respiratory infection.

^a^ GINA Global Strategy for Asthma Management and Prevention 2017 (available from www.ginasthma.org)

### 3.3 Asthma treatment and monitoring

Table 3 summarizes patients’ asthma treatment and monitoring. 81.7% of adults and 64.4% of children reported having used controller medications in the last 12 months. However, only 42.4% and 29.8% of respective groups reported being compliant to the treatment. Less than 1 in 7 adults and 1 in 3 children had AAP discussed, however, only less than half of them had used it.

**Table 3:** Asthma treatment and monitoring

	Adults (*N* = 1092)	Children (*N* = 188)
	*n* (%)	*n* (%)
On controller medications	892 (81.7)	121 (64.4)
Reported Noncompliant^a^	514 (57.6)	85 (70.2)
Scheduled follow‐up	772 (70.7)	131 (69.7)
Average frequency of follow‐up in the past 12 months	4	3
Had peak flow rate measured in the last 3 visits	597 (54.7)	101 (53.7)
Has asthma Diary	426 (39.0)	94 (50.0)
Recorded most times	142 (33.3)	35 (38.0)
Had education on Asthma Action Plan (AAP)	150 (13.7)	52 (27.7)
Had used AAP	56 (38.4)	28 (53.8)

Abbreviations: *N*, denominator; *n*, numerator

^a^ Noncompliant is defined as patient self‐reported of missing controller medications in the last one month.

### 3.4 Asthma education

More than 90% of the patients had received asthma education. However, the emphasis on specific education varied; education on inhaler technique was the usual focus, whereas the importance of asthma diary and AAP was less emphasized.

### 3.5 Asthma control and its associated factors

Tables 4 and 5 show variables significantly associated with poorly controlled asthma. For adults, variables that were put into the stepwise logistic regression model (*p* < 0.25) included gender, ethnicity, source of health payment, distance by road (in kilometers) to nearest healthcare facility, ex‐smoker, co‐morbidities, triggers (upper respiratory tract infection, haze, fumes, food, extreme emotions, cold weather, air‐conditioning, and physical activity), emergency visits, use of and compliance to controller medications, use of CAM, provision of AAP, and education on inhaler technique. For children, variables that were put into the models (*p* < 0.25) included pre‐mature birth, education level, distance in kilometers to nearest healthcare facility, passive smoker, use of and compliance to controller medications, follow‐up, triggers (upper respiratory tract infection and hot weather), allergic rhinitis, provision of AAP, and asthma diary, and education on asthma. In the final model, factors significantly associated with poorly controlled asthma in adults were self‐reported exposure to haze (OR 1.51; 95% CI 1.13–2.01); food (OR 1.54; 95% CI 1.15–2.07); extreme emotion (OR 1.90; 95% CI 1.26–2.89); air‐conditioning (OR 1.63; 95% CI 1.20–2.22); physical activity (OR 2.85; 95% CI 2.13–3.82); emergency visit at least once in the past year (OR 1.76; 95% CI 1.33–2.33); received education on inhaler technique (OR 2.29; 95% CI 1.48–3.56), not using CAM (OR 1.76; 95% CI 1.24–2.49), and being male (OR 1.74; 95% CI 1.28–2.36). In addition, with every unit kilometer increase in the distance between patients' home and the nearest healthcare facility increases the odds of poor control by 5% (OR 1.05; 95% CI 1.01–1.10) as well as in patients whose main source of health payment was from personal savings, the odds of poor control increased as compared to employer‐subsidized health payments (OR 1.71; 95% CI 1.21–2.43). In children, significant associated factors were hot weather (OR 3.14; 95% CI 1.22–8.11); allergic rhinitis (OR 2.57; 95% CI 1.13–5.82); on controller medications (OR 2.54; 95% CI 1.27–5.09) and given asthma action plan (AAP) (OR 2.20; 95% CI 1.05–4.65).

**Table 4:** Odds ratios and 95% confidence intervals in the final model after stepwise model selection for poor asthma control in adults

Variable	Odds ratio	95% CI	*p*‐value
Gender (male)	1.74	1.28–2.36	<0.001
Source of health payment (employer)			0.011
Personal	1.71	1.21–2.43	0.003
Government	1.43	0.90–2.27	0.129
Distance (kilometer) to nearest healthcare facility	1.05	1.01–1.10	0.013
Haze as asthma trigger	1.51	1.13–2.01	0.005
Cold food as asthma trigger	1.54	1.15–2.07	0.003
Extreme emotion as asthma trigger	1.90	1.26–2.89	0.002
Air‐conditioning as asthma trigger	1.63	1.20–2.22	0.002
Physical activity as asthma trigger	2.85	2.13–3.82	<0.001
Had emergency visit at least once in past 12 month	1.76	1.33–2.33	<0.001
Education on inhaler technique	2.29	1.48–3.56	<0.001
Non‐CAM user	1.76	1.24–2.49	0.001

Abbreviations: CAM, complementary alternative medicine; CI, confidence interval.

**Table 5:** Odds ratios and 95% confidence intervals in the final model after stepwise model selection for poor asthma control in children

Variable	Odds ratio	95% CI	*p*‐value
On controller medications	2.54	1.27–5.09	0.008
Hot weather as asthma trigger	3.14	1.22–8.11	0.018
Allergic rhinitis	2.57	1.13–5.82	0.024
Given AAP by health care provider	2.20	1.05–4.65	0.038

Abbreviations: AAP, asthma action plan; CI, confidence interval.

## 4 DISCUSSION

### 4.1 Summary of findings

We enrolled a total of 1280 adults and children with asthma in a cohort to assess their asthma control and to identify associated risk factors and health care they received. The majority of the patients’ asthma control was still poor. Although 90% reported having been given asthma education, less than a quarter had been given asthma self‐management advice. Most patients had regular reviews and were prescribed controller medications, however, only half reported being compliant. In adults, key associations with poor control were history of emergency visits, triggers such as food, emotion, air‐conditioning, haze, and exercise, as well as socio‐demographic characteristics such as male gender, distance from healthcare facility, lack of employer‐subsidized health payment, and not using CAM. Received education on inhaler technique, taking controller medications and given AAP were associated with poor control, which likely reflected targeting of care.

### 4.2 Interpretation in relation to other literature

The prevalence of suboptimal asthma control was consistent with findings of previous studies; 34% of adults in different health clinics in Selangor, Malaysia,^27^ and 18% in studies in Southeast Asia.^28^, ^29^, ^30^ We found that self‐reported exposure to haze, air‐conditioning, extreme emotion, and physical activity were significantly associated with poor control in adults. A probable reason could be adults are more likely to engage in work‐related activities, either indoor, or outdoor. While avoiding these triggers whenever possible is important, action plans should include advice for stepping‐up controller medications if patients encounter triggers, for example, during the haze or when doing physical activity.^31^

In contrast, hot weather and allergic rhinitis were risk factors for poorly controlled asthma in children. A recent review determining the relation between ambient temperature and exacerbations of asthma in children had reported extreme hot or cold were common triggers.^32^ It is believed that this occurred in children with eosinophilic endotype, the hypothesis being that hot and humid weather (as in Malaysia) facilitates microorganisms or mites to grow in the respiratory tract.^33^, ^34^

The importance of addressing the link between rhinitis and asthma has been highlighted in the World Health Organization (WHO) report and recommended in several guidelines.^35^, ^36^ In this study, allergic rhinitis was associated with poorly controlled asthma in children but not in adults, although the proportion of patients with allergic rhinitis is similar. Possible reasons for this difference include the severity of allergic rhinitis, and undertreated (or untreated) allergic rhinitis in children. A study on children with asthma and allergic rhinitis reported that only a third received proper rhinitis treatment.^37^ One observational study reported the association of poor controlled asthma and allergic rhinitis was only in children who were not on treatment or only on oral antihistamines/montelukast compared to those who used nasal corticosteroids.^38^

The association of the male gender with poorly controlled asthma has been reported in several studies.^39^, ^40^ Whilst this may reflect different phenotypes of asthma, it may also be due to differences in exposure to triggers or in attitude towards treatment. Earlier studies had reported relation to men's health‐seeking behavior and attitude towards adherence to asthma treatment in particular the inhaled corticosteroids (ICS).^41^, ^42^

In this study, we noted that among those who did not use CAM, the odds of having poor control were 1.76 times higher than CAM users, indicating a link between the use of CAM and good asthma control. However, it is not clear if this is because of the therapeutic effect of the CAM or the behavior of CAM users. Those with well‐controlled asthma might favor maintaining CAM use while those with poor control might view CAM as ineffective and switched to evidence‐based medicine. A local qualitative study reported use of traditional or herbal medicine, homeopathy, and supplements to combat symptoms of asthma^43^, ^44^ and those who used CAM were observed to be more proactive towards their asthma self‐management.^43^ However, the effectiveness of CAM remains unclear. A survey in Canada showed that CAM use was associated with poorly controlled asthma, but a recent systematic review showed no clear conclusion was reached.^45^, ^46^

We showed that evidence‐based interventions were associated with increased odds for poor control; taking controller medications, education on inhaler technique, and provided AAP for asthma self‐management may represent ‘confounding by indication,’ as healthcare providers appropriately targeted care on the higher‐risk individuals. Confounding by indication occurs because clinicians are more likely to provide evidence‐based interventions to patients with more severe disease, this resulted with recommended treatments appear to link with poorer outcomes.^47^ For example, our findings that controller medications and action plan ownership were associated with an increased risk of acute attacks in children (despite robust evidence that they both prevent exacerbations), have been identified as confounding by indication in other studies.^48^, ^49^

In our study, the prescription of controller medications (mainly ICS) seemed appropriate (82% in adults and 64% in children in the last 12 months). However, only about half of the patients reported being compliant with this treatment. A similar trend was seen across the Asia‐Pacific region, where the use of ICS remains low despite the implementation of national guidelines on ICS recommendations.^50^, ^51^ We found about half of the adults and a quarter of the children reported use of SABA more than twice a week. Patients may rely on SABA for quick‐acting relief instead of the delayed clinical benefits provided by ICS, leading to the underuse of controller medications.^52^ Overuse of SABA is significantly associated with asthma morbidity and mortality.^53^ This highlights a worrying trend, and education on asthma medications is the key to improve the situation.

The use of asthma diary and AAP in this population was suboptimal. Less than a third of these patients had been given AAP despite extensive evidence‐based reviews concluding that asthma self‐management supported by regular healthcare professional review improves asthma control, and reduces exacerbations and admissions.^54^ This is similar to findings in the Asthma Insights and Reality in Asia‐Pacific (AIRIAP) study in which only 18% of the respondents had been given a written action plan and this did not take into account whether the action plan was used or used effectively.^50^, ^55^ Supported self‐management of asthma should be emphasized in primary care practice. A pictorial asthma action plan or mobile applications on self‐management tailored to patients' or carers' needs could be explored to improve compliance to medication and self‐monitoring.^56^, ^57^, ^58^, ^59^

The prevalence of smoking in people with asthma have been reported to be similar to the general population.^50^ We found a relatively low prevalence of current smokers, 9.5% in adults and 1.6% in children, compared to the Malaysian national average of 21.3% among adults.^16^ It is worrying that almost half of our patients reported being exposed to second‐hand smoke; in children, fathers were the main source. Passive smoking is a recognized risk factor for poor asthma control.^60^ Secondhand smoke can be reduced if the family members are made aware of the risks. The Malaysian government had implemented smoke‐free legislation in public spaces, a step towards protecting the public from second‐hand smoke.^61^

Obesity has been linked with poor asthma control^62^, ^63^ and is not described as a phenotype of asthma.^22^ We did not show any significant association between obesity and asthma control, although there was a high proportion of overweight and obese patients.

### 4.3 Strengths and limitations

The strength of this study was the wide age range of patients who participated (5–87 years old). The proportion of children was relatively low, which may not represent the actual population in the Klang District. Missing data was minimal as the questionnaires were administered face‐to‐face. Most variables were self‐reported (trigger factors, EQ‐VAS, frequency of asthma exacerbation, asthma care they received, and treatment compliance), which could lead to response bias. However, some information was retrieved from clinical records to limit the bias. Recruitment was limited by convenience sampling of patients attending the clinic for asthma follow‐up, which was reflected by the findings that 70% of patients received scheduled care. This might not include patients who chose not to attend the health clinics for their routine care who may have better, or worse control. A limitation of the cross‐sectional design of this study is that it cannot demonstrate causality, and three of our observed associations with poor controlled asthma were likely to be due to ‘confounding by indication.’

### 4.4 Implications for future practice and research

The findings of this study have highlighted that further improvement in primary care practice should be carried out to improve asthma control. A personalized approach to asthma care should be encouraged. The association of various triggers with poor control highlights the need for a personalized consultation. Reasons for noncompliance with controller medications should be explored and addressed individually. Implementation of supported self‐management of asthma should be the focus in routine asthma care in Malaysia. Further studies should evaluate an intervention that can address and facilitate the delivery of personalized care and supported self‐management of asthma, for example, a simplified care plan for health care providers. Public policy on environmental pollution (e.g. haze) could be developed to help minimize asthma triggers.

## 5 CONCLUSION

Our study revealed that the status of well‐controlled asthma is yet to be achieved in these patients. Although this study was limited by its convenience sampling, the baseline findings have provided important input to inform a better strategy in improving patients' asthma status. Several identified triggers deserve attention during asthma reviews. Non‐adherence to controller medications despite a good rate of prescription, frequent use of SABA, and underutilized asthma self‐management needed to be re‐emphasized to improve and sustain better asthma outcomes.

## AUTHOR CONTRIBUTIONS

**Norita Hussein**: Conceptualization; data curation; formal analysis; funding acquisition; investigation; methodology; project administration; resources; supervision; validation; visualization; writing – original draft; writing – review & editing. **Su May Liew**: Conceptualization; data curation; formal analysis; investigation; methodology; project administration; resources; supervision; validation; visualization; writing – review & editing. **Nik Sherina Hanafi**: Conceptualization; data curation; formal analysis; investigation; methodology; project administration; resources; supervision; validation; visualization; writing – review & editing. **Ping Yein Lee**: Conceptualization; data curation; formal analysis; investigation; methodology; project administration; resources; supervision; validation; visualization; writing – review & editing. **Ai Theng Cheong**: Conceptualization; data curation; formal analysis; investigation; methodology; project administration; resources; supervision; validation; visualization; writing – review & editing. **Sazlina Shariff Ghazali**: Conceptualization; data curation; formal analysis; investigation; methodology; project administration; resources; supervision; validation; visualization; writing – review & editing. **Karuthan Chinna**: Conceptualization; data curation; formal analysis; investigation; methodology; software; supervision; validation; writing – review & editing. **Yong Kek Pang**: Conceptualization; data curation; formal analysis; investigation; methodology; project administration; resources; supervision; validation; visualization; writing – review & editing. **Asiah Kassim**: Conceptualization; data curation; formal analysis; investigation; methodology; project administration; resources; supervision; validation; visualization; writing – review & editing. **Richard A. Parker**: Data curation; formal analysis; investigation; methodology; software; supervision; validation; writing – review & editing. **Jürgen Schwarze**: Conceptualization; data curation; formal analysis; methodology; project administration; resources; supervision; validation; visualization; writing—review & editing. **Aziz Sheikh**: Conceptualization; data curation; funding acquisition; methodology; resources; supervision; validation; visualization; writing – review & editing. **Hilary Pinnock**: Conceptualization; data curation; funding acquisition; investigation; methodology; resources; supervision; validation; visualization; writing – review & editing. **Ee Ming Khoo**: Conceptualization; data curation; formal analysis; funding acquisition; investigation; methodology; project administration; resources; supervision; validation; visualization; writing – review & editing.

## CONFLICT OF INTEREST STATEMENT

Ee Ming Khoo, Ping Yein Lee, Ai Theng Cheong, Hilary Pinnock and Aziz Sheikh report grants from the National Institute for Health Research (NIHR) Global Health Research Unit on Respiratory Health (RESPIRE). Ee Ming Khoo reports personal fees from AstraZeneca; and is the President of the International Primary Care Respiratory Group and the Primary Care Respiratory Group Malaysia. Yong Kek Pang is the President of the Malaysian Thoracic Society and advisory committee for Novartis, AstraZeneca and Boehringer Ingelheim. Aziz Sheikh reports on grants from Asthma UK and HDR UK. All other authors declare no conflicts of interests. The National Institute for Health Research (NIHR) Global Health Research Unit on Respiratory Health (RESPIRE) funded this study, however, there is no direct involvement of decision in study design; collection, analysis, and interpretation of data; writing of the report except needing their approval to report for publication. For other financial bodies, there is no involvement. All authors have read and approved the final version of the manuscript. Honorary Professor Dr. Ee Ming Khoo (corresponding author) has full access to all of the data in this study and takes complete responsibility for the integrity of the data and the accuracy of the data analysis. The views expressed in this publication are those of the author(s) and not necessarily those of the NIHR or the UK Department of Health and Social Care.

## TRANSPARENCY STATEMENT

The lead author Ee Ming Khoo affirms that this manuscript is an honest, accurate, and transparent account of the study being reported; that no important aspects of the study have been omitted; and that any discrepancies from the study as planned (and, if relevant, registered) have been explained.

## Data Availability

The data that support the findings of this study are available from the corresponding author upon reasonable request.
